# LeFood-set: Baseline performance of predicting level of leftovers food dataset in a hospital using MT learning

**DOI:** 10.1371/journal.pone.0320426

**Published:** 2025-05-19

**Authors:** Yuita Arum Sari, Atsushi Nakazawa, Yudi Arimba Wani

**Affiliations:** 1 Graduate School of Interdisciplinary Science and Engineering in Health Systems, Okayama University, Kita-Ku, Okayama, Japan; 2 Faculty of Computer Science, Brawijaya University, Malang, Indonesia; 3 Nutrition Department, Faculty of Health Sciences, Brawijaya University, Malang, Indonesia; University of Georgia, UNITED STATES OF AMERICA

## Abstract

Monitoring the remaining food in patients’ trays is a routine activity in healthcare facilities as it provides valuable insights into the patients’ dietary intake. However, estimating food leftovers through visual observation is time-consuming and biased. To tackle this issue, we have devised an efficient deep learning-based approach that promises to revolutionize how we estimate food leftovers. Our first step was creating the LeFoodSet dataset, a pioneering large-scale open dataset explicitly designed for estimating food leftovers. This dataset is unique in its ability to estimate leftover rates and types of food. To the best of our knowledge, this is the first comprehensive dataset for this type of analysis. The dataset comprises 524 image pairs representing 34 Indonesian food categories, each with images captured before and after consumption. Our prediction models employed a combined visual feature extraction and late fusion approach utilizing soft parameter sharing. Here, we used multi-task (MT) models that simultaneously predict leftovers and food types in training. In the experiments, we tested the single task (ST) model, the ST Model with Ground Truth (ST-GT), the MT model, and the MT model with Inter-task Connection (MT-IC). Our AI-based models, particularly the MT and MT-IC models, have shown promising results, outperforming human observation in predicting leftover food. These findings show the best with the ResNet101 model, where the Mean Average Error (MAE) of leftover task and food classification accuracy task is 0.0801 and 90.44% in the MT Model and 0.0817 and 92.56% in the MT-IC Model, respectively. It is proved that the proposed solution has a bright future for AI-based approaches in medical and nursing applications.

## Introduction

Observation of patients’ leftover food is a daily task in hospitals since it can evaluate patients’ nutritional intake. By analyzing patients’ leftovers, healthcare professionals can obtain valuable insights into the patient’s health status [1], including malnutrition [2] and depression [3]. Excessive leftovers can not only be a barrier to cost-saving measures but also negatively impact patients’ recovery rates. Thus, it is essential to improve the quality and taste of meals in nutrition facilities to reduce food waste and improve patient outcomes [4]. In a hospital context, analyzing meal leftovers give significant information regarding numerous areas of the hospital’s food service [5]. Based on a food waste survey conducted at a regional public hospital in Sidoarjo, Indonesia, there is a high residual rate of patient food, above the standard of 20% Limited meal variation, variances in inpatient classes, a lack of diversity in menu items, and patients not touching the same food due to surgery all contribute to this high residue. As a result, there is a massive loss of food that the patients did not consume [6]. Hospitals also assess patient satisfaction with meals delivered by studying food leftovers [7]. Thus, recording and evaluating leftovers in hospital and long-term care settings is an important task in terms of quality of care and environment. It is also important for the general consumer market.

Human visual estimation simplifies the weighing process, which is called subjective evaluation. Dietitians or nutritionists in hospital settings rely on qualified assessors to weigh food or perform visual examinations such as Comstock level analysis [8, 9]. Comstock level prediction is widely used in food consumption surveys to assess leftover food. This approach is the most popular and used in hospitals [10]. However, it not only requires a meticulous, skilled and trained estimator, but is also prone to frequent overestimation or underestimation, resulting in uncertainty about the actual food intake of patients or others. Based on these backgrounds, several researchers have leveraged artificial intelligence (AI) in an attempt to minimize the estimation errors in visual observations [11–13]. This could serve as a decision support tool for dietitians and nutritionists estimating food leftovers of hospitalized patients.

In this paper, we propose a method to solve this problem by deep learning-based image recognition. Specifically, we have developed a method to estimate the amount of food eaten with higher accuracy than human observation, simply by inputting images before and after serving. Existing studies used simple image processing techniques such as counting areas using image segmentation; however, the method had large measurement errors [14]. In recent years, deep learning has been used in research on areas such as daily food consumption, improving bodily health, and reducing the occurrence of diseases. These universal methods can be applied to various domains without explicit feature extraction, allowing for extensive application in food computing, especially through image recognition for nutrition analysis [15]. [11] used convolutional neural-network (CNN) to estimate the amount of leftover liquid meals in hospitals. In deep learning projects, VGG and ResNet as examples of CNN models are often used as baselines. VGG architecture primarily comprises convolutional layers utilizing 3x3 filters along with max-pooling layers, illustrating that enhancing its depth can lead to better performance overall [16]. ResNet architecture help address the vanishing gradient issue in deep neural networks. These pathways enable information to traverse through layers, enhancing the network’s learning capabilities. By preserving or boosting performance, ResNets are capable of achieving a greater depth compared to VGG networks [17]. VGG networks are commonly used as a standard for comparison due to their high performance in image classification tasks [18]. On the other hand, ResNet is also frequently used as a benchmark in experiments designed to test the scalability of new topologies or their effectiveness in deeper networks [17]. The CNN model has not yet been applied to a limited dataset with imbalanced data in each food category to predict leftover foods objectively. Therefore, in this study, a multitasking model was implemented to calculate leftover food. Against this background, this study has the following highlights:

1. The first large-scale open dataset for leftover food prediction was presented. The dataset contains 524 pairs of images from 34 food categories showing the food portions before and after consumption. In addition, there is data on the observed amount of leftover food and the results of weighing using digital scales both before and after consumption.
2. A novel method for predicting leftovers and food categories using deep learning, which outperforms human observation.
3. In an experiment, we implemented and compared several network architectures. As a result, the methods using multi-task (MT) learning architecture performed better than that of not using MT learning.

The remainder of this paper is organised as follows. [sec:related_work]Sect 1 outlines the related research on this topic. In [sec:dataset]Sect 2, a detailed description of the dataset is presented. [sec:method]Sect 3 explains methodology of this research. In [sec:results]Sect 4, the experimental results used as a baseline are presented. A discussion is provided in [sec:discussion]Sect 5, and limitations are elaborated in [sec:limitations]Sect 6. Finally, the article is concluded in [sec:conclusion]Sect 7.

## 1. Related work

Relevant literature on image-based food analysis and leftover prediction is reviewed in this section. Aizawa *et al*. [19] proposed an approach that uses images to obtain individual dietary patterns to improve food balance estimation. Their food recording system included food image extraction [20], food balance analysis [21], and summarization and visualization [22]. Food photos act as input to identify categories and weights in the nutritional evaluation system developed in [23]. Shape templates and area-based weights are used to increase the accuracy of the system [21, 24, 25]. Several methods showed the algorithm of calculating the number of food regions pixel-wise or segmented-based algorithms [26]. [26–28] attempted to estimate food waste from images of plates before and after a meal, taking into account background variations and the need for background removal. The algorithm identified utensils as part of the plate during and after a meal, resulting in an estimation inaccuracy of 45.8%. Hence, due to the inherent limitations in accuracy and adaptability of the pixel-wise method in leftover calculation, an alternative strategy is required.

Recently, deep learning has been used in research in areas such as [11] that compares the accuracy of estimating liquid food residue using computer vision. The study focuses only on liquid foods and provides recommendations for future research to determine whether AI-based measurement techniques can be readily used by medical professionals in clinical settings, as the usefulness of the proposed AI-based measurement method is currently unknown. The AI estimation approach had lower errors than the visual estimation approach for fermented milk, peach juice, and total. However, there was no significant difference in errors for thin rice gruel. Errors in AI estimation were biased towards leftovers. Overall, AI estimation had smaller mean squared errors and higher coefficient of determination values for fermented milk and peach juice compared to visual estimation. Total *R*2 value was equal in accuracy for both AI and visual estimations.

Another research uses a deep learning object identification network to identify food categories and calculate meal consumption levels in pre- and post-meal photographs. The method uses mask R-CNN to identify food categories and homography transformation to correct post-meal images based on meal plate regions. Meal intakes are estimated by comparing pre-meal and post-meal images of measured food volumes. However, this method is limited by the definition of the shape of the food in the container in 3D shapes, whether a spherical cap, cone, or cuboid [29].

Other studies connected to artificial intelligence are also utilized for measuring food intake [30]. A smartphone-based system named FoodIntech uses AI to recognize and calculate food leftovers without human intervention. The system uses images of a QR code on the meal tray to detect and recognize food items. Testing showed the method to be reliable, with excellent results for 39% of dishes and good results for 19%. Implementing this method can improve dish recognition and can be expanded with more photos to include new dishes and foods. This study employs deep learning techniques for the segmentation of areas. However, the calculation of food waste is based on pixel-wise resolution, which presents a limitation. The accuracy of the pixel-wise calculation heavily relies on the quality of the segmentation of food areas.

The hospital can utilize various datasets based on its specific requirements and different methodological approaches to address this issue. The primary focus of this research is on analyzing remaining food from 2D images, enabling the predictive learning model to accurately predict leftovers and recognize different types of food simultaneously. We encountered challenges when searching for a comparable dataset that includes information on food consumption before and after specific weight measurements and leftovers, particularly within a hospital environment. A paper closely related to our research has been previously published by [11]. However, the dataset in the study mainly focuses on liquid foods, such as 432 servings of rice, 72 servings of fermented milk, and 72 servings of peach juice. Unfortunately, this dataset is not available for public use, preventing us from utilizing it for future research and development.

## 2 Dataset

This chapter describes the details of the dataset we have constructed. The dataset can be accessed at the following link: https://data.mendeley.com/datasets/cchsk79jkt/1.

### 2.1 Data acquisition

**Fig 1:**
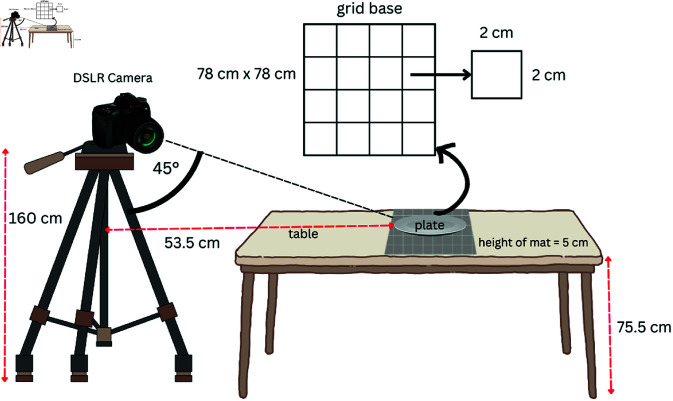
Setting up the environment for data acquisition.

Images were taken at a hospital in Malang, Indonesia, using a Nikon D3200 DSLR camera. The focus of the study was a single food item with solid and liquid components. Solid and liquid foods were served on plates and in bowls, respectively. White dishes were used throughout. To photograph the food, we used a camera, tripod, external flash, table, wooden placemat, reflector, and sheet of paper as illustrated in Fig 1. The food was presented on a 78×78cm wooden placemat with a black and white checkerboard pattern, with each grid measuring 2×2cm. The placemat was placed on a table measuring 80 cm in length, 80 cm in width, and 75.5 cm in height to ensure optimal and consistent plate or bowl placement during data collection. The camera was set as follows: F = 4.5, ISO 100, exposure = -2, manual flash mode with REPEAT 1/128, wide camera range, AF-2 focus mode, and positioned at a 45-degree angle to the diagonal meeting point of a placemat. The camera was then mounted on a tripod at maximum height and positioned 53.5 cm from the diagonal meeting point of a placemat. We used a white cloth reflector stretched on three sides of the placemat opposite the camera to improve the image quality. The data consisted of images of meals taken before and after consumption and the difference in weight obtained by physical measurements. The weight was measured by weighing the plate containing food using a digital scale.

### 2.2 Dataset annotation

The dataset comprises 524 image pairs before and after consumption. For each image pair, the following information is annotated as shown in Table 1.

**Table 1:** The detail of the dataset showing the ID, food name, the number of example pairs in total and each food leftover level given by a human observer, where the leftover level is ranging from 1 (Not consumed at all) to 7 (zero remaining).

ID	Name	Total	Leftover level (human obs.)						
			1	2	3	4	5	6	7
1	Bubur (*Porridge*)	1	1	0	0	0	0	0	0
2	Nasi (*Rice*)	78	14	1	7	8	11	9	28
3	Tim (Rice Porridge)	76	9	1	8	14	9	8	27
4	Telur Orak Arik (*Scrambled Eggs*)	1	1	0	0	0	0	0	0
5	Kroket Daging (*Meat Croquettes*)	19	2	0	0	0	2	1	14
6	Opor Ayam (*Chicken Braised in Coconut Milk)*	2	1	0	0	0	0	0	1
7	Opor Telur (*Opor Eggs*)	2	1	0	0	0	0	1	0
8	Ikan Acar Kuning (*Yellow Pickled Fish*)	20	6	1	1	2	2	1	7
9	Bali Putih Telur (*Bali Egg White*)	4	0	0	0	1	0	2	1
10	Bali Telur (*Bali Eggs*)	21	5	0	0	2	0	1	13
11	Ayam Bumbu Srundeng (*Srundeng Seasoned Chicken*)	9	3	0	0	1	1	1	3
12	Rendang Ayam (*Chicken Rendang*)	18	3	1	2	0	1	4	7
13	Ayam Bumbu Laos (*Galangal Seasoned Chicken*)	16	7	1	1	1	1	2	3
14	Ayam Bumbu Bistik (*Steak Seasoned Chicken*)	22	3	0	0	3	2	5	9
15	Ikan Bb Asam Manis (*Sweet and Sour Seasoned Fish*)	14	0	1	2	2	0	2	7
16	Telur Mata Sapi (*Sunny Side Up*)	20	3	0	0	1	2	2	12
17	Ayam Suwir (*Shredded Chicken*)	5	0	0	1	0	1	0	3
18	Oseng Bakso (*Stir Fry Meatballs*)	5	0	0	0	2	0	1	2
19	Oseng Sosis (*Stir Fry Sausage*)	4	1	1	0	0	0	0	2
20	Sambel Goreng Rempela Ati (*Sambal Goreng Rempela Ati*)	15	9	0	0	0	0	2	4
21	Bola Bola Daging (*Meat Balls* )	6	0	0	0	1	0	0	5
22	Tumis Bakso (*Stir Fry Meatballs*)	12	1	2	0	0	0	1	8
23	Dadar Jagung Goreng *(Fried Corn Omelet*)	6	0	0	0	1	0	0	5
24	Bali Tahu (*Balinese Tofu*)	22	10	2	0	0	0	0	10
25	Perkedel Kentang (*Potato Cakes*)	22	3	0	0	2	1	0	16
26	Tahu Goreng (*Fried Tofu*)	1	0	0	0	0	0	0	1
27	Tahu Bumbu Kecap (*Tofu Soy Sauce*)	19	4	3	2	3	0	1	6
28	Tempe Bumbu Kuning (*Yellow Seasoning Tempeh*)	3	0	0	0	1		1	1
29	Tempe Goreng (*Fried Tempe*)	6	3	0	0	0	0	0	3
30	Tempe Bumbu Bacem (*Seasoned Tempeh*)	28	6	1	1	1	1	2	16
31	Tahu Bulat (*Round Tofu*)	11	5	0	0	0	1	1	4
32	Orek Tempe (*Orek Tempeh*)	22	3	0	0	5	2	2	10
33	Dadar Jagung Kukus (*Steamed Corn Omelet*)	3	2	0	0	0	1	0	0
34	Oseng Tahu (*Oseng Tofu*)	11	1	2	2	3	1	1	1

**Fig 2:**
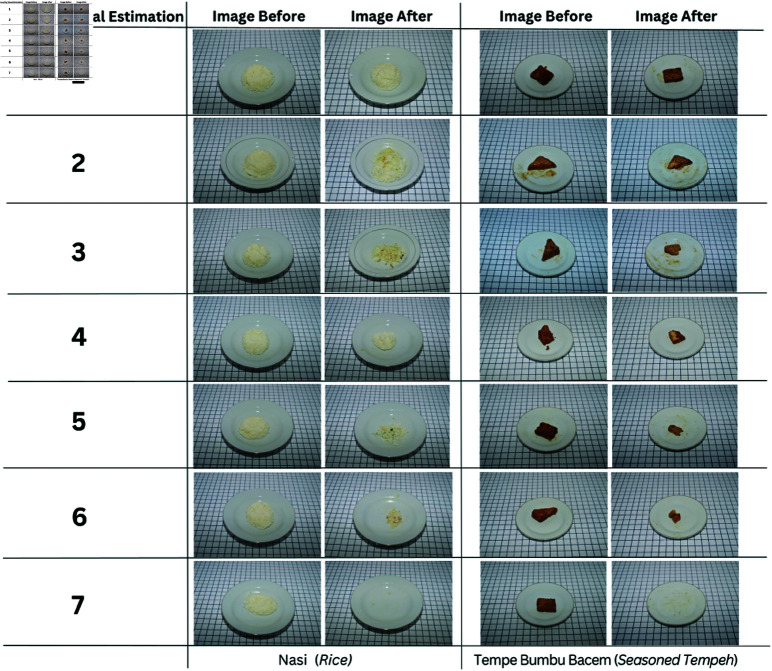
Example of pictures taken before and after eating based on seven different levels of leftovers.

**id** The IDs for the food category.
**name** The name of the food.
**weight_before** The weight of meal before eaten (gram as unit measurement).
**weight_after** The weight of meal after eaten (gram as unit measurement).
**visual observation of leftover** Level of leftover given by a trained observer or skilled trainer based on visual estimation ranging from 1 (Not consumed at all) to 7 (zero remaining). Samples for each level are presented in Fig 2.

### 2.3 Dataset analysis

**Fig 3:**
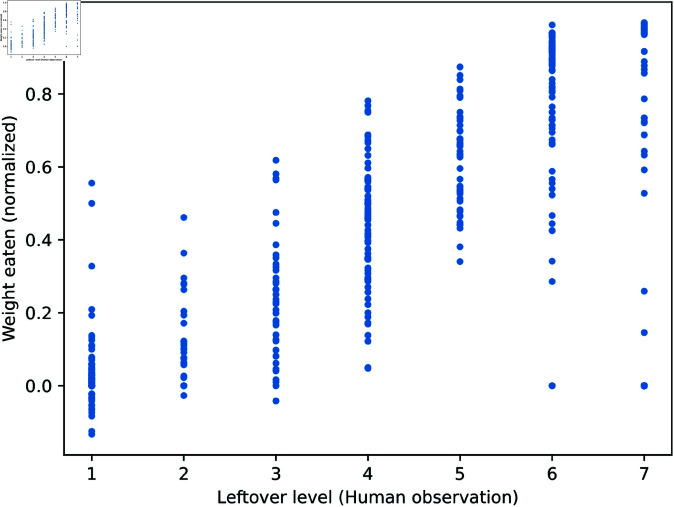
The relation of leftover levels of human visual observation (x-axis) and weight (y-axis), where the Peason’s correlation r = 0.95.

Fig 3 and Table 2 show the correlation between the leftover level (human observation) and the weight eaten (normalized from 0 to 1). As seen, a strong correlation was found between the two data; namely, the Pearson correlation *r* between them is 0.95. It is also found that the standard deviations of the weights in levels 1 and 7 as described in Fig 2 are relatively smaller than those in levels 2 to 6. These statistics suggest a larger difference in judgments when the observer predicted at the intermediate levels.

**Table 2:** The relation between leftover levels and weight.

Leftover level	7	6	5	4	3	2	1
weight (a.v.)	0.029	0.153	0.270	0.436	0.620	0.813	0.978
weight (S.D.)	0.11	0.20	0.20	0.17	0.13	0.17	0.09

## 3. Method

### 3.1 Network architectures

**Fig 4:**
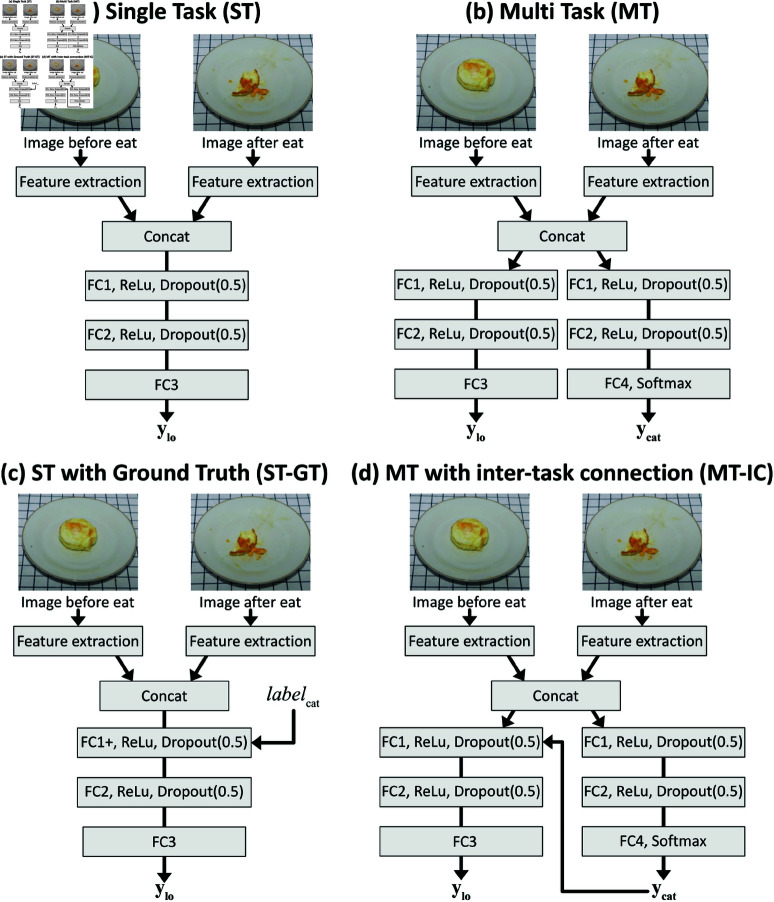
Network architectures. (a) Single task (ST) model, (b) Multit-ask (MT) model, (c) Single task model with Ground Truth food category (ST-GT), and (d) Multi-task model with inter-task connection (MT-IC). The feature extraction layers are pre-trained CNN, including VGG11, VGG13, VGG16, VGG19, ResNet50, ResNet101, or ResNet152. FC1, FC2, FC3, FC4, and FC1+ are fully connected layers whose input and output dimensions are (4096, 1024), (1025, 512), (512, 1), (512, 50), and (4096+34, 1024), respectively. *y*_*lo*_ and *y*_*cat*_ are the normalized leftover level (0.0 - 1.0) and food category, and *label*_*cat*_ in the model (c) is the label of the ground-truth food category.

We designed four end-to-end convolutional neural networks to infer the amount of food consumed from two images (before and after eating). Fig 4 shows the network structures. In all architectures, image features are extracted by feature extraction networks, concatenated, and transferred to fully connected (FC) network(s).

The single-task (ST) model is a naive regression model for leftovers, and the multi-task (MT) model outputs the amount of leftovers and the food category simultaneously. The single-task model with ground truth (ST-GT) is a model assumed to be used when the food category is given. Namely, the ground truth food category is combined to the first fully connected layer. The multi-task model with inter-task connection (MT-IC) is designed to provide feedback on the inference result of the food category and is explicitly used for leftover estimation. A model with food classification as ground truth was also incorporated, designated as the MT model with Food Category Ground Truth Feedback (MT-GT). This model is employed for the general evaluation of performance in comparison to the MT-IC model. Fundamentally, food classification in the MT-GT model is determined based on the provided label, which is expected to result in a lower error rate compared to the MT-IC model.

### 3.2 Loss function and training

Regarding the leftover value, the networks are trained to output the leftover value normalized from 0.0 to 1.0. Assuming the output of the network as *y*_*lo*_, the loss function *L*_*lo*_ is given as in Equation 1,

$$L_{lo}=\left|y_{lo}-\frac{\text{weight\_before}-\text{weight\_after}}{\text{weight\_before}}\right|$$ (1)

Regarding the inference of food category, the loss function *L*_*cat*_ is given as the binary cross entropy, namely, as stated in Equation 2

$$L_{cat}=\text{binary\_cross\_entropy}(y_{cat},label_{cat})$$ (2)

where *y*_*cat*_ and *label*_*cat*_ are the inference result and the label of the food category.

In training MT models (MT and MT-IC), both losses are linearly combined as Equation 3:

$$L_{mt}=\alpha \times L_{lo}+(1-\alpha )\times L_{cat}$$ (3)

In the implementation, we set α to 0.9. The models are optimized using the Adam optimizer with *l*_*r*_ set to 0.0001. Early stopping was used to stop training after the first 20 consecutive epochs if performance did not improve. The primary goal of early stopping was to monitor the model’s performance on a separate validation dataset that was different from the training dataset. The data augmentation used included random horizontal flip, random vertical flip, random rotation, random padding, random Gaussian blur, random sharpness adjustment, and random contrast with a probability of 1/7.

## 4 Results

The data were divided into three sets: training, validation, and test sets. We used 7/10 for training, 2/10 for validation, and 1/10 for testing. Each model was tested in 10-fold cross-validation and took their average.

### 4.1 Leftover level prediction

**Fig 5:**
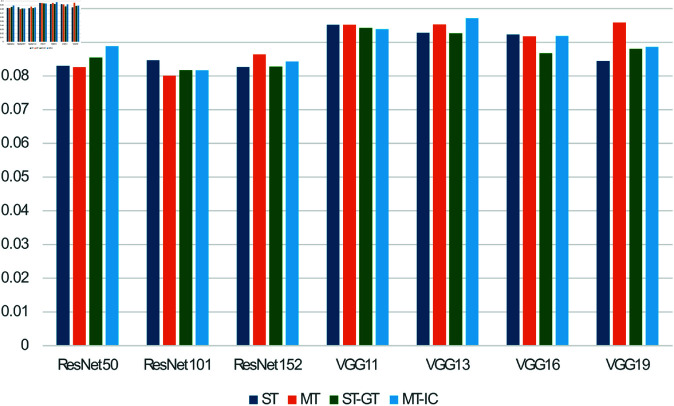
Result of food level estimation (mean absolute error (MAE), smaller is better).

Table 3 and Fig 5 shows the mean absolute error (MAE) of leftover prediction of all models (ST, ST-GT, MT, and MT-IC) with different feature extraction networks (ResNet50, Resnet101, ResNet152, VGG11, VGG13, VGG16, and VGG19). For the reference, the MAE of human visual observation (Fig 2) was computed assuming the visual leftover level 1,...,7 as 1/7, 2/7, 3/7, ..., 1.0 and computed the MAE to the normalized weight values. Overall, the ResNet group outperformed the VGG group in MAE and classification accuracy. Regarding the network architecture, MT model outperformed others and human visual observation, namely, MAE of human observation is 0.0926, while the MT model, MT-GT and MT-IC with ResNet101 are 0.0801, 0.07826 and 0.0816, respectively.

**Table 3:** Result of leftover level estimation (MAE (S.D)).

Feature extraction	ST	MT	ST-GT	MT-IC
ResNet50	0.0830 (0.017)	0.0826 (0.017)	0.0854 (0.019)	0.0888 (0.019)
ResNet101	0.0846 (0.022)	**0.0801** (0.020)	**0.0817** (0.014)	**0.0817** (0.018)
ResNet152	**0.0826** (0.021)	0.0863 (0.019)	0.0828 (0.018)	0.0843 (0.019)
VGG11	0.0952 (0.022)	0.0952 (0.017)	0.0942 (0.027)	0.0939 (0.015)
VGG13	0.0928 (0.016)	0.0953 (0.018)	0.0926 (0.018)	0.0971 (0.022)
VGG16	0.0923 (0.022)	0.0918 (0.021)	0.0868 (0.020)	0.0919 (0.019)
VGG19	0.0844 (0.020)	0.0958 (0.020)	0.0880 (0.017)	0.0886 (0.021)
Human observation	**0.0926**			

**Fig 6:**
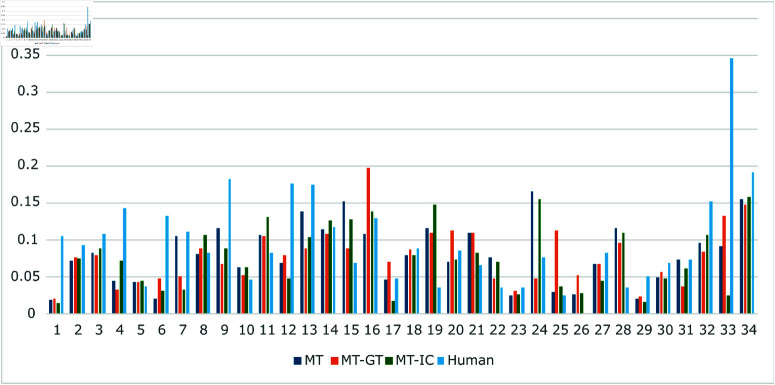
Class-wise MAE (smaller is better). *x*-axis shows the food ID.

Fig 6 shows the MAE of each food category. This allows the tendency of the food category results towards the food leftover prediction results to be determined. Twenty-two food categories, representing 34 categories overall, demonstrate that the MT model outperformed human observation. Meanwhile, the MT-GT and MT-IC models are better in 20 food categories compared to humans. This indicates that the proposed MT model can accurately predict the majority of food leftover levels.

### 4.2 Food category classification

**Fig 7:**
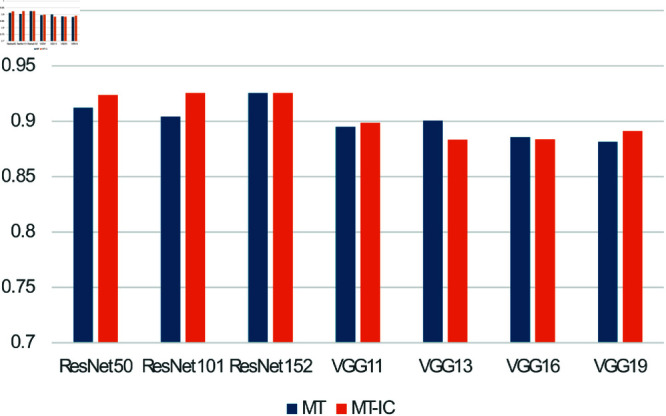
Result of food classification accuracy (higher is better).

Table 4 and Fig 7 show the accuracy of food classification. Similar to the leftover level prediction, ResNet groups outperformed VGG groups, namely, the accuracy reached 92.56% when ResNet152 were used to MT and MT-IC.

**Table 4:** Result of food classification.

Feature extraction	MT	MT-IC
ResNet50	0.912 (0.034)	0.924 (0.025)
ResNet101	0.904 (0.055)	**0.926** (0.041)
ResNet152	**0.926** (0.039)	**0.926** (0.029)
VGG11	0.895 (0.038)	0.899 (0.054)
VGG13	0.901 (0.043)	0.884 (0.032)
VGG16	0.886 (0.044)	0.884 (0.043)
VGG19	0.882 (0.023)	0.891 (0.047)

## 5 Discussion

In general, the proposed method, comprising the ST, ST-GT, MT, and MT-IC Models utilizing ResNet101, exhibits a smaller error than a human observer’s visual estimation. The incorporation of weight information derived from food classification automatically from the fully connected layer (MT-IC Model) has also been observed to enhance the precision of estimates, as evidenced by a reduction in standard deviation values compared to the MT Model.

**Fig 8:**
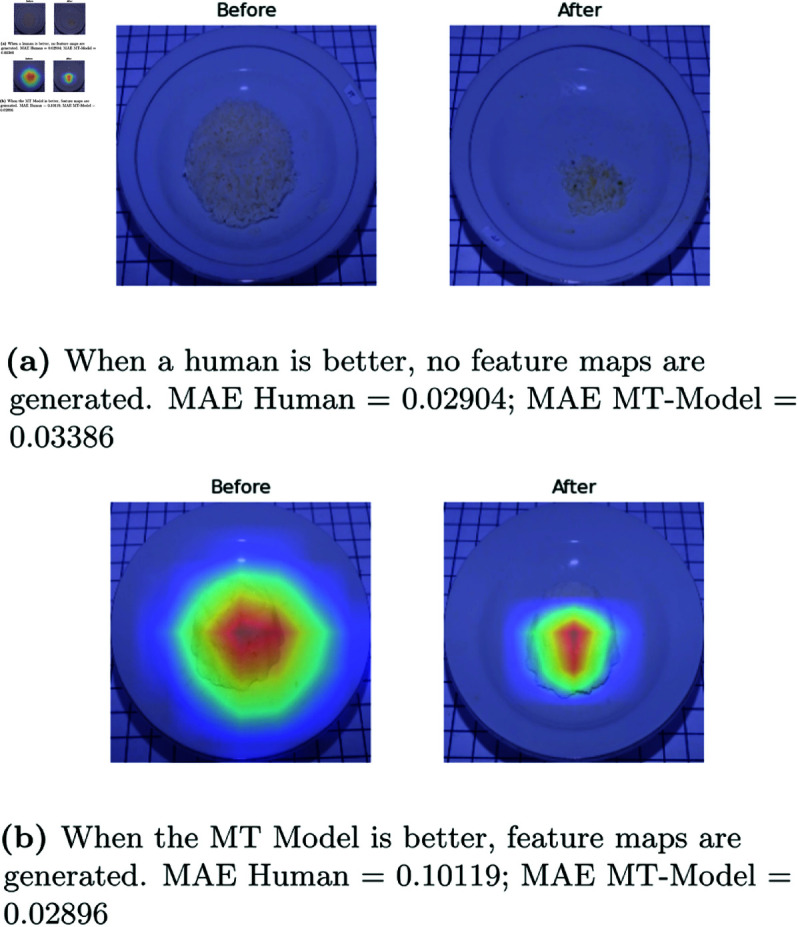
Visualization of leftover prediction in MT Model vs Visual Estimation by Human Observer.

**Proposed v.s Human observation.** The results of the analysis were subjected to further investigation through the use of a visual explanation, which elucidates the significance of feature extraction. The results of explainable visualization are represented in GradCAM [31]. In most of the cases, the proposed algorithm grasped the features of the food. However, in instances, it could not extract features and observed wrong portions, such as empty regions when human observers were superior, as illustrated in Fig 8.

**Fig 9:**
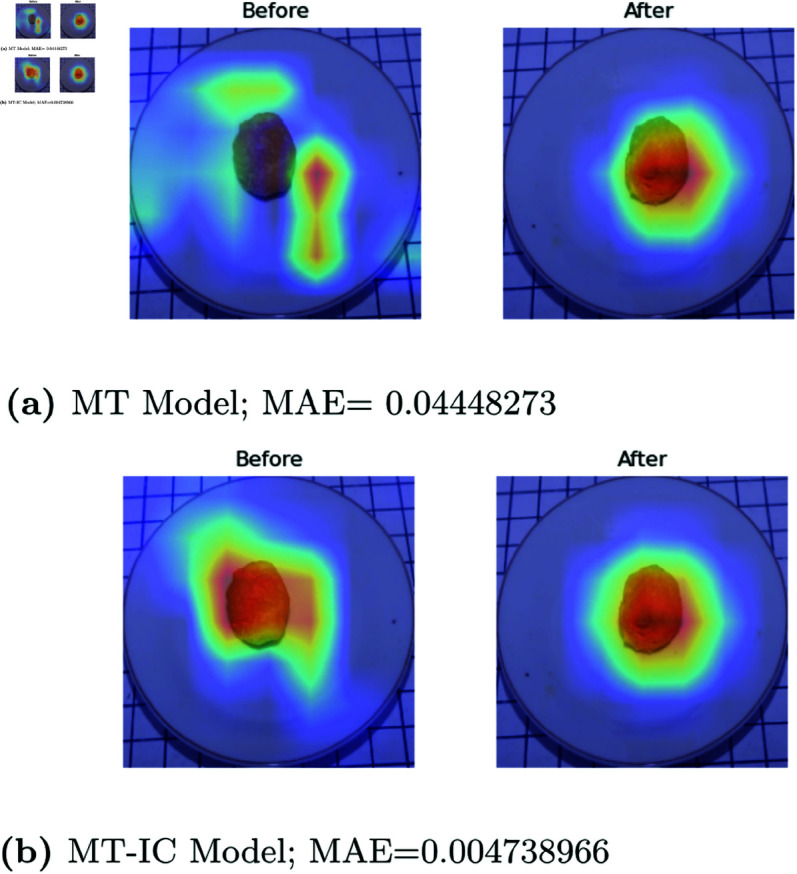
GradCAM of leftover prediction in MT Model and MT-IC Model of Tahu Bulat (Round Tofu)).

**Methods comparison.** Incorporating food type information into the regression model to predict leftovers can improve the effectiveness of the regression procedure. This is evidenced by Fig 9, which illustrates that GradCAM with the MT-IC Model (see Fig 9(b)) can effectively delineate the object. In contrast, the GradCAM feature maps in the MT Model (see Fig 9(a)) also cover plates and tables in the before image, which impairs the success of feature extraction. This also indicates that, in the presence of such conditions, the MAE of the MT Model is greater than the MT-IC Model. The impact of incorporating food type information from the food classification task into the leftover prediction regression task is also evident in the GradCAM results. These results are corroborated by the MAE value, which indicates that GradCAM visualizations effectively identify objects having a lower MAE than those that are unable to perform feature extraction correctly.

**Fig 10:**
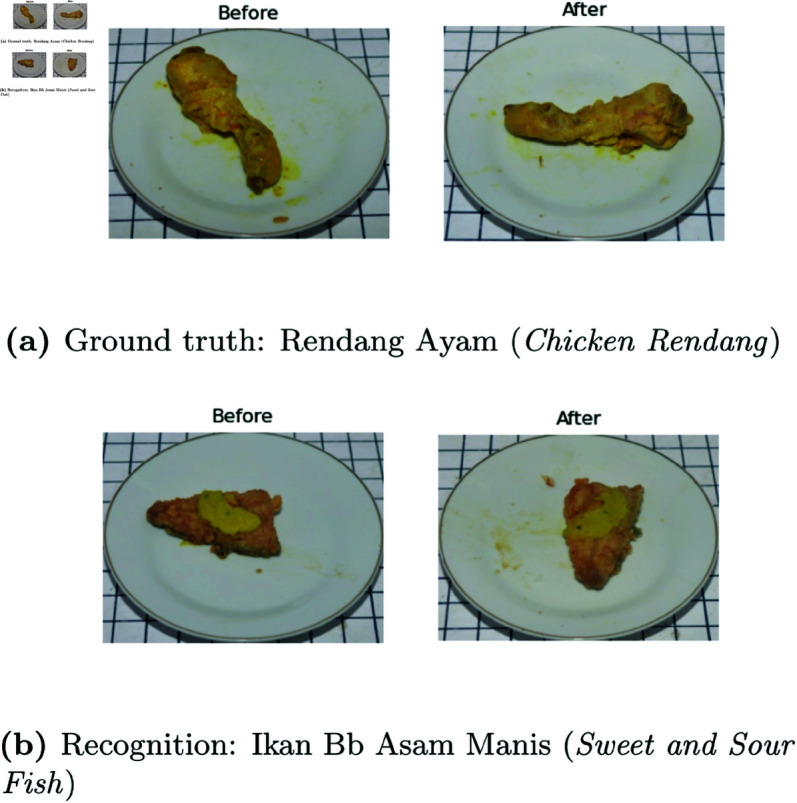
Incorrect food classification.

**Food Classification.** Overall, the accuracy of food classification based on experimental findings in multi-task learning is notably high, exceeding 90%. In this instance, MT-IC has a competitive edge over MT by incorporating food classification data into the regression function to predict leftovers. Fig 10 demonstrates that errors in food classification can occur when items belonging to various categories share resemblances in terms of both color and shape.

**Fig 11:**
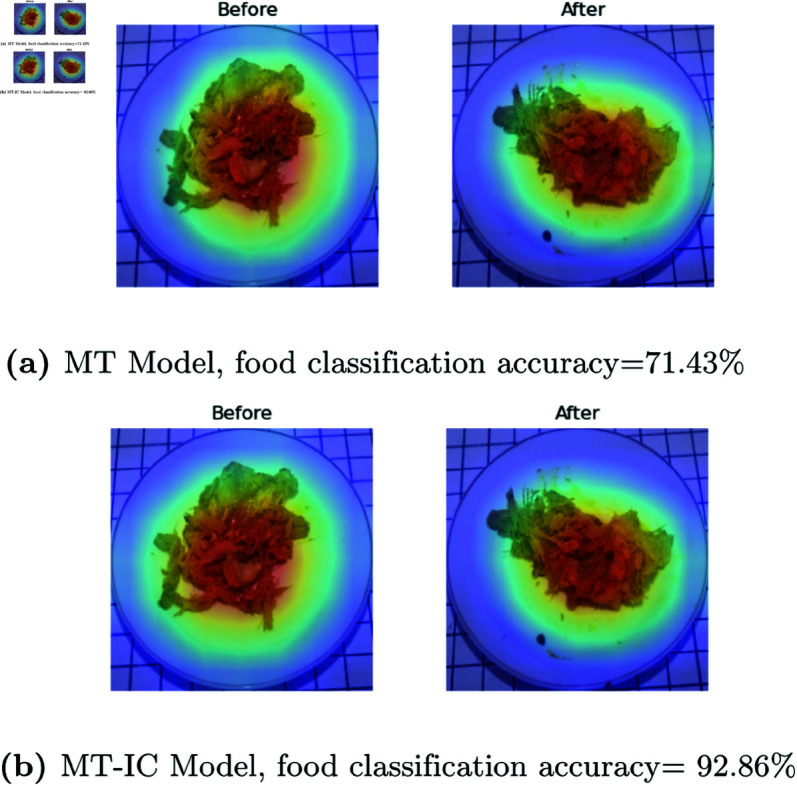
Impact in Food Classification Task: GradCAM of leftover prediction in MT Model, and MT-IC Model of 12.Ayam Bumbu Laos (Galangal Seasoned Chicken).

Adding class information into regression also has positive impacts on classification tasks, including feature extraction. In Fig 11, for food type 12, that is Ayam Bumbu Laos (*Galangal Seasoned Chicken*), the feature map of MT-IC Model matches the shape of the object, as well as MT-Model. In that model, the accuracy of the MT-IC Model is 92.86% while the MT Model achieves 71.43%. Performance on food classification is somehow affected by the number of data. The correlations between accuracy and the number of data for the MT Model and MT-IC Model are 0.511 and 0.480, respectively. The correlations also show that the MT-IC Model has a lower dependence on data size compared to the MT Model. On the other hand, the number of data has less impact on food estimation results, indicated by the correlation of 0.061 and 0.163 for the MT Model and MT-IC Model, respectively.

## 6 Limitations

**Fig 12:**
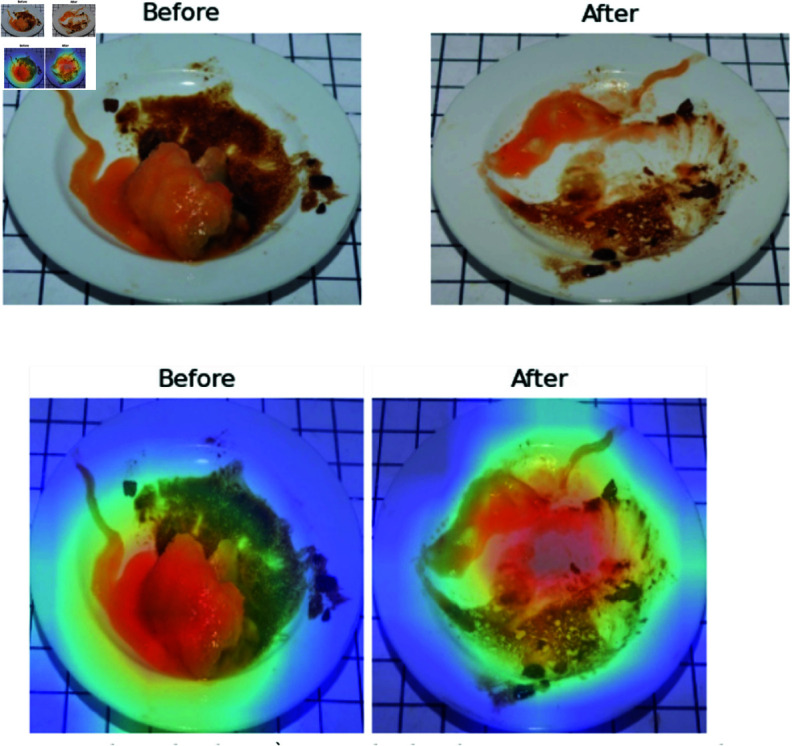
Failure cases (too oily dishes). While humans are observed to be fully consumed, the proposed methods detect the source (oil) as the food of leftover.)

The proposed method is susceptible to error when predicting leftovers that contain excessive oil or a minimal amount of sauce (see Fig 12). The predictions of human observers indicate that the food has been completely consumed (100% consumed). In contrast, the multi-task learning models (MT Model and MT-IC Model) indicate that there is still food remaining on the plate. This is evidenced by GradCAM failing to generate a heatmap in the leftover prediction task, indicating that no features are extracted effectively to predict leftovers. Despite the failure of the leftover task to predict leftovers, the food recognition task demonstrates the capacity to identify the type of food accurately.

**Fig 13:**
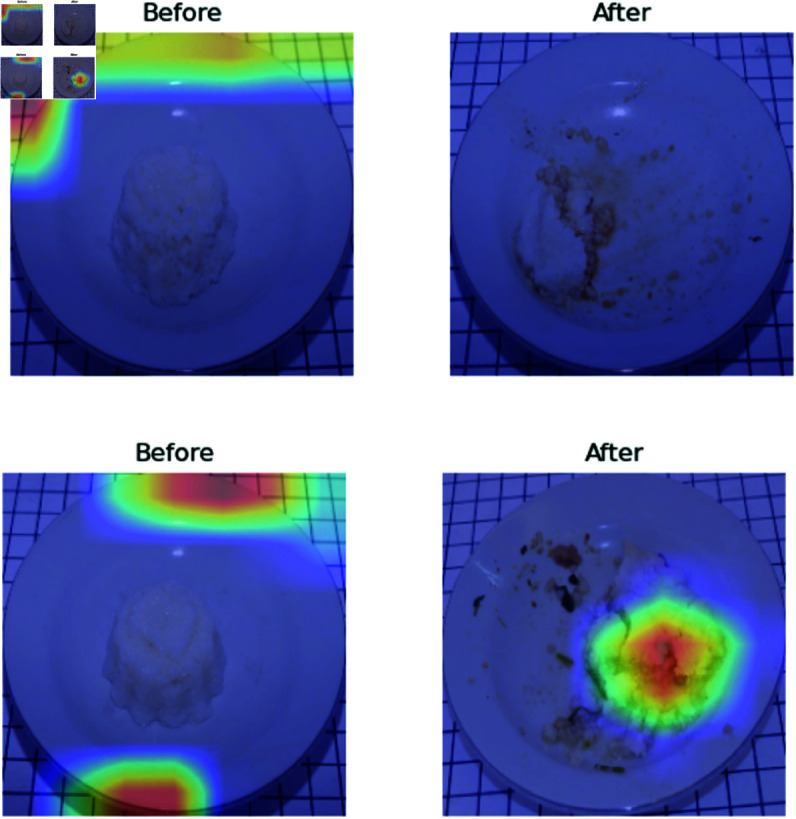
Failure case of extracting feature in rice (above) and rice porridge (bottom).

In instances involving images of rice and rice porridge, human predictions typically outperform computer algorithms due to the difficulties in accurately extracting features from the objects. The generated heatmap does not emphasize the object itself but focuses on the surrounding areas, including post-consumption changes undetectable by GradCAM (see Fig 13). This discrepancy is largely attributed to the similarity in color between the background and the object, thus complicating the analysis process.

In addition, the large number of sample data has an impact on the performance of the proposed model. Ideally, a larger amount of data will lead to more accurate predictions than a smaller amount of data. However, in this study, the sample data used was imbalanced in several food categories, which resulted in inconsistencies in the number of samples. In practice, this phenomenon occurs in hospital settings where patients may receive different types of menus or foods based on their individual dietary needs. The quantity of food in certain categories may be limited due to the patient’s diet. While this research addresses the issue, further enhancements to the model or performance of the proposed architecture can be achieved by increasing the number of samples through image sample generation techniques. However, the concern is not only for generating the images but also the information information attached to this data, such as information on the weight of food before and after consumption.

## 7 Conclusion

This paper introduces a novel dataset featuring images captured before and after food consumption. The dataset is augmented with subjective evaluations of the leftover food by an observer and objective weight measurements using digital scales commonly found in hospitals. The research findings illustrate the capability of utilizing the AI approach that uses deep learning techniques to predict the amount of leftover food both in subjective and objective evaluation. Overall, the MT Learning approach is superior to human visualization estimation for predicting leftover amounts. We propose two multi-task learning models: the MT and the MT-IC models. The optimal results were achieved using ResNet101, with MAE of 0.0801 for the leftover task and 90.44% for the food classification accuracy task in the MT Model and 0.0817 for the leftover task and 92.56% for the food classification accuracy task in the MT-IC Model, respectively. In general, the MT Model is more effective than the ST Model. The MT-IC Model is a model in which the ground truth food category information is automatically extracted, thereby enabling the MT-IC Model to be automatically fed from the food recognition task. The MT-IC Model is more precise than the MT Model, as evidenced by the smaller standard deviation value observed in the MT-IC Model. In addition, MT-IC is also effective for data sets with a limited number of samples.

This paper is not without its shortcomings. Firstly, The presence of oil or sauce in image data disrupts the detection of objects that are then estimated. This can lead to discrepancies between the assessment made by humans and that made by the proposed model, with the latter classifying the objects as food waste. Secondly, feature extraction remains an ongoing challenge when dealing with objects and backgrounds of the same color. Lastly, the data is imbalanced across food categories and levels of leftovers, which negatively impacts the accuracy of leftover predictions.

In future studies, it will be valuable to enlarge the dataset and investigate multiple potential applications, including food recognition, food image segmentation, food image retrieval, food image generation, and other techniques that can precisely forecast leftover foods. Further research is required in the field of nutritional analysis in hospitals using this dataset as an alternative for decision support. Additional data pertaining to the composition of food ingredients are necessary to predict the nutritional needs of patients.
